# Supplementary material for: Isolation, identification, and antimicrobial susceptibility pattern of Campylobacter jejuni and Campylobacter coli from cattle, goat, and chicken meats in Mekelle, Ethiopia
Source: PLoS One. 2021 Feb 10;16(2):e0246755. doi: 10.1371/journal.pone.0246755 (PMC7875392; doi:10.1371/journal.pone.0246755)
Supplement: Attachment — Submitted filename: Response to Reviewers.docx [file pone.0246755.s001.docx]

**Manuscript PONE-D-20-31123**

**Response to Reviewers**

**Dear Publisher**

Thank you for allowing us to submit a revised draft of the manuscript “**Isolation, Identification, and Antimicrobial Susceptibility Pattern of *Campylobacter jejuni* and *Campylobacter coli* from Cattle, Goat, and Chicken Meats in Mekelle, Ethiopia**” with an opportunity to address the reviewers’ comments for publication in your esteemed journal, PLOS ONE.

Moreover, we would like to say **You** and both **Reviewers** many thanks for your invaluable time, critical and professional evaluation, as well as dedication to providing feedback on our manuscript, and are grateful for the insightful comments on and valuable improvements to our manuscript. We have a great appreciation for the corrections given by the reviewers and found them more valuable and professional for the betterment of our manuscript.

Before we proceed to the response to the reviewers, let us explain the overall content of our manuscript and Journal requirement issues.

1. Initially, we have prepared our manuscript according to the PLOS ONE style templates.
2. We didn’t take Figure 1 (Administrative Map of Mekelle City) from other sources rather it is generated by us, however, we have removed it from the manuscript. Similarly, the image of Figure 2 (PCR Products: M-DNA ladder; Lane 1-4 *C. coli* (462bp); Lane 6-11 *C. jejuni* (589bp); and Lanes 5 and 12-Negative control) was taken by us.
3. In the revised manuscript, we have included/referred to Table 5 in the text.

We have incorporated most of the suggestions made by the reviewers though we have seen that the majority of comments require how and why explanations. Those changes are highlighted within the Revised Manuscript with Track Changes. Please see below, for a point-by-point response to the reviewers’ comments and concerns:

1. We have made some grammatical corrections as **Reviewer 2** mentioned.
2. As **Reviewer 1** stated the samples that were collected from the different meat types seem to be non-systematic and have a great variation among them. The reason for the variation in the number of samples collected from the different species of animals is that due to the accessibility/availability of the respected samples. Hence, the majority of bovine are slaughtered at abattoirs. However, the majority of goat and chicken are slaughtered at the home level without the recognition of the concerned bodies of the City and are most of the time inaccessible for researchers. **Reviewer 1** also suggested us to indicate the numbers of samples collected from each source, hence we have included under the sample size and sample collection section of the manuscript.
3. **Reviewer 1** mentioned that the ladder for PCR is not well defined and bands are not clear. It is quite right that the bands' resolution is not that much, however, we have mentioned under the PCR section of the methodology as each band of the ladder has a molecular weight difference of 100bp. The samples were collected from different sources and we run the whole 64 phenotypically positive samples, hence, Figure 2 is a representative.
4. There were differences in hygienic conditions and evisceration of the internal organs which lead to cross-contamination.
5. **Reviewer 1** mentioned why the highest prevalence is found in poultry? It is a well-known fact that poultry appeared to be a significant source of *Campylobacter* and chicken were found to be heavily intestinal carriers of *Campylobacter* when compared with other food animals in addition to what the reviewer suggested, sample size.
6. As per **Reviewer 1’s** comment, we have made corrections in the reference section.
7. **Reviewer 2** needs an explanation for the small sample size of chicken meat which is also raised **by Reviewer 1**. So, we have indicated above in no. 2.
8. **Reviewer 2** indicated that as there is an editorial problem in measurement, we have corrected it as per the given comment.
9. **Reviewer 2** needs an explanation for the flow of meat till it reaches the restaurant. As per our Country’s practice, the animals are first transported to the abattoir to be slaughtered, and then the meat is either directly taken to the restaurant or the butcher shops depending upon the customer. As it is presented in Table 1, the highest prevalence was recorded in meat samples collected from restaurants.
10. **Reviewer 2** indicated that some words and numbers are cut off. So, we have corrected them. However, related to the number outside of the bracket it is not 2 rather it is 62, which means out of the 64 isolates 62 (96.9) had shown resistance against Ampicillin.
11. **Reviewer 2** needs an explanation of how sample size variation affects the prevalence estimation. In epidemiologic studies, the sample size has an important role to detect an effect and to achieve the desired precision in estimates of the parameter of interest. A small sample size will not provide a precise estimate and reliable answers to the study hypothesis. Hence, sample size variation may lead to a foregone conclusion in the prevalence of a disease.
12. **Reviewer 2** has also asked the potential sources of Campylobacter in cattle and goat meat. It is known that *Campylobacter species* are normally carried in the intestinal tracts of many domestic livestock such as poultry, cattle, sheep, goat, pigs, as well as wild animals and birds. Fecal matter is a major source of contamination and could reach carcasses through a direct deposition. The meat of cattle and goat origin can become contaminated by this pathogen during slaughtering and carcass dressing.
13. **Reviewer 2** has requested us to elaborate on the possible reasons for variations in the prevalence of Campylobacter in the different sources of samples. It is an interesting comment. This might be because of an extra chance of acquiring contamination in restaurants due to mishandling of meat or cross-contamination among different carcasses or individuals who are working in restaurants might serve as a source of contamination.
14. **Reviewer 2** requested us to explain about the two genes. Several multiplex PCR assays have been used to detect *Campylobacter spp*., *C. coli,* and *C. jejuni*. Most of these assays have used a variety of species-specific genes such as *omp50*, 16S rRNA, 23S rRNA, *hipO*, *mapA*, *ceuE* or putative aspartokinase for identification of *C. coli* and *C. jejuni*. The two commonly used species differentiation markers are *mapA* and *ceuE* . A species-specific 24-kDa membrane-associated protein of *C. jejuni*, named MAPA for membrane-associated protein is encoded by the *mapA* gene, which is useful for genetic identification of *C. jejuni* by PCR. *ceuE* encodes a 34.5 to 36.2 kDa lipoprotein component of a binding-protein-dependent transport system for the siderophore enterochelin. In the current study, for *C. jejuni*, the *mapA* gene was used and for *C. coli*, the gene *ceuE* was used.
15. Reviewer 2 has recommended us to include the timeline of antibiotic resistance as well as the potential routes of exposure of Campylobacter to antibiotics in the various regions. Since we didn’t see the current study as per the given comment, we can’t do anything.
