# Supplementary material for: Isolation, identification, and antimicrobial susceptibility pattern of Campylobacter jejuni and Campylobacter coli from cattle, goat, and chicken meats in Mekelle, Ethiopia
Source: PLoS One. 2021 Feb 10;16(2):e0246755. doi: 10.1371/journal.pone.0246755 (PMC7875392; doi:10.1371/journal.pone.0246755)
Supplement: Attachment — Submitted filename: Response to Reviewers.docx [file pone.0246755.s002.docx]

**Manuscript PONE-D-20-31123**

**Response to Reviewers**

**Dear Publisher**

Thank you for allowing us to submit the revised draft of the manuscript “**Isolation, Identification, and Antimicrobial Susceptibility Pattern of *Campylobacter jejuni* and *Campylobacter coli* from Cattle, Goat, and Chicken Meats in Mekelle, Ethiopia**” with an opportunity to address the reviewers’ comments for publication in your esteemed journal, PLOS ONE, for the second time.

Moreover, we would like to say **You** and both **Reviewers** many thanks for your invaluable time, critical and professional evaluation, as well as dedication to providing feedback on our manuscript, and are grateful for the insightful comments on and valuable improvements to our manuscript. We have a great appreciation for the corrections given by the reviewers and found them more valuable and professional for the betterment of our manuscript.

We have incorporated almost all of the comments given by the reviewers. Those changes are highlighted within the Revised Manuscript with Track Changes. Please see below, for a point-by-point response to the reviewers’ comments and concerns:

1. We have made the corrections given at Line 69, Line 79, Line 87, Line 191-192, Line 219-223, Line 270-273, and Line 283-298 as per the comments.
2. Regarding the comment related to the image in Figure 1, we have agreed with the reviewers’ idea. However, as we have already indicated in our previous response to reviewers, we can’t replace the figure with a new one since by the time the gel-doc was not working properly and we had taken the picture using our mobile phone’s camera. So, we have decided either to keep it as it is or to remove it from the manuscript. If you believe that as it is better to remove it, we don’t have a reservation on it. Hence, you can do that.
